# Recent Advances in Wearable Optical Sensor Automation Powered by Battery versus Skin-like Battery-Free Devices for Personal Healthcare—A Review

**DOI:** 10.3390/nano12030334

**Published:** 2022-01-21

**Authors:** Nikolay L. Kazanskiy, Muhammad A. Butt, Svetlana N. Khonina

**Affiliations:** 1 Samara National Research University, 443086 Samara, Russia; kazanskiy@ipsiras.ru (N.L.K.); khonina@ipsiras.ru (S.N.K.); 2 IPSI RAS-Branch of the FSRC “Crystallography and Photonics” RAS, 443001 Samara, Russia; 3 Institute of Microelectronics and Optoelectronics, Warsaw University of Technology, Koszykowa 75, 00-662 Warszawa, Poland

**Keywords:** wearable sensors, skin-like, heart rate monitoring, continuous glucose monitoring, battery-free sensors

## Abstract

Currently, old-style personal Medicare techniques rely mostly on traditional methods, such as cumbersome tools and complicated processes, which can be time consuming and inconvenient in some circumstances. Furthermore, such old methods need the use of heavy equipment, blood draws, and traditional bench-top testing procedures. Invasive ways of acquiring test samples can potentially cause patient discomfort and anguish. Wearable sensors, on the other hand, may be attached to numerous body areas to capture diverse biochemical and physiological characteristics as a developing analytical tool. Physical, chemical, and biological data transferred via the skin are used to monitor health in various circumstances. Wearable sensors can assess the aberrant conditions of the physical or chemical components of the human body in real time, exposing the body state in time, thanks to unintrusive sampling and high accuracy. Most commercially available wearable gadgets are mechanically hard components attached to bands and worn on the wrist, with form factors ultimately constrained by the size and weight of the batteries required for the power supply. Basic physiological signals comprise a lot of health-related data. The estimation of critical physiological characteristics, such as pulse inconstancy or variability using photoplethysmography (PPG) and oxygen saturation in arterial blood using pulse oximetry, is possible by utilizing an analysis of the pulsatile component of the bloodstream. Wearable gadgets with “skin-like” qualities are a new type of automation that is only starting to make its way out of research labs and into pre-commercial prototypes. Flexible skin-like sensing devices have accomplished several functionalities previously inaccessible for typical sensing devices due to their deformability, lightness, portability, and flexibility. In this paper, we studied the recent advancement in battery-powered wearable sensors established on optical phenomena and skin-like battery-free sensors, which brings a breakthrough in wearable sensing automation.

## 1. Introduction

**Figure 1:**
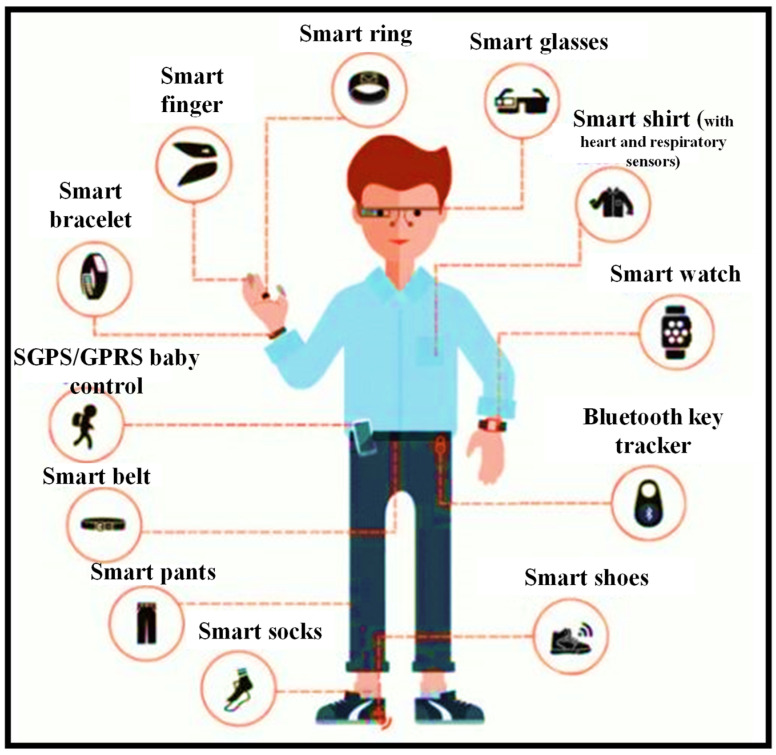
Wearable sensors can be worn almost anywhere on the body for daily activities, including health monitoring [12].

Miniaturization advances have led to several wearable sensors that are now being employed in a variety of biomedical applications [1,2]. Some of these have been ingrained in people’s daily lives [3]. Smart bands and smart watches with pulse monitors, pulse oximeters, accelerometers, gyroscopes, and other sensors are one example [4,5,6]. Implantable devices have been utilized since the 1950s when the first pacemaker was utilized to bring back a regular heart rhythm by continually pumping only the ventricle [7]; since then, implantable automation has evolved to include the most modern implants, for instance Ocular and Cochlear implants [8,9]. Even though biomedical implants are vital to the advancement of Medicare, they frequently necessitate the assistance of a trained specialist [10]. Wearables, on the other hand, are more intelligible and may be employed without the need for medical or technical competence [11]. Their purpose is to harvest data without requiring any surgical processes or the insertion of materials that are more likely to cause long-term negative effects. These sensors can be worn anywhere one wants to wear them. While this sounds like an exaggeration, with sensors implanted in clothing and other form factors in addition to wrist-worn devices, it is principally true. Figure 1 shows the rise of wearables and the future of wearable technology [12].

The application of wearable devices and arrangements to accomplish immediate recognition of variations in patient status necessitating therapeutic intervention is a rising topic of study in the field of wearables. The care of patients with chronic obstructive pulmonary disease (COPD) is one instance of this sort of wearable use. Early diagnosis of exacerbation events is an important objective in the therapeutic care of patients with COPD. Exacerbations, which are described as bouts of augmented dyspnea, cough, and a variation in the volume and type of sputum, are a regular occurrence in the natural course of COPD, and they can cause functional impairments and disability. Exacerbations should be detected and treated as soon as possible to avoid worsening clinical conditions and the need for emergency department care or hospital admission. Remote monitoring devices can aid in the early detection of patterns in patient’s health state that indicate an impending exacerbation. One way to address the problem of early identification of exacerbation episodes is to monitor variations in patient’s level of activity and presume that a drop in activity level indicates the possibility of a deterioration of the individual’s clinical state without further monitoring [13,14].

Atallah et al. have created an ear-worn sensor that may be employed to track activities and degrees of effort in patients with COPD [15]. The researchers were able to recognize numerous different types of physical activities, as well as the intensity of those activities, using powerful machine learning techniques and a single ear-worn sensor. Steele et al. [16] and Belze et al. [17] measured human movement in 3D over three days and found that the extent of the acceleration vector verified in patients with COPD was correlated with measures of patient status, such as the six-minute walk distance, the FEV1 (Force Expiratory Volume in One Second), the severity of dyspnea, and the physical function domain of the health-related quality of life scale. Hecht et al. [18] proposed an algorithm established on data gathered using a single unit for a minute-by-minute study of patients’ activity levels. The method was put to a test in 22 patients for 14 days. The scientists also employed a simple empirically created algorithm to assess whether the person was wearing the gadget, allowing them to keep track of compliance. Another noteworthy finding from the same study was that during the first few days of assessment, individuals tended to increase their activity level. This finding shows that, monitoring should be performed regularly to prevent noticing the transient effects caused by the fact that the subject is aware that he or she is being watched.

Wearable sensing automation has quickly evolved from a science fantasy concept to a wide range of well-established user and medical devices [19]. The affordability and user-friendly arrangement offered by developments in miniaturized electronics, the development of smartphones and connected devices, an increasing consumer wish for health awareness, and the unmet prerequisite for medical practitioners to uninterruptedly acquire medical quality data from their patients are all contributing to the expansion of wearable sensors [20]. Despite initial success, there is still a thirst for even more data from the body. Most of the sensing modalities, including in current wearables (pulse, galvanic skin response, etc.) are non-specific, hence this desire remains unmet (e.g., how many factors can increase one’s pulse or trigger one to sweat). Additionally, most wearable sensor devices use techniques that have been around for years. Even the most complex wearables, such as continuous transdermal glucose monitors, benefit from over 30 years of advancements in enzyme electrodes discovered in basic and ultra-low-cost finger-prick glucose test strips. Transdermal glucose assessment is, in fact, possibly the only widely employed wearable sensor that continuously monitors the state of a serious condition (diabetes) [21,22].

**Figure 2:**
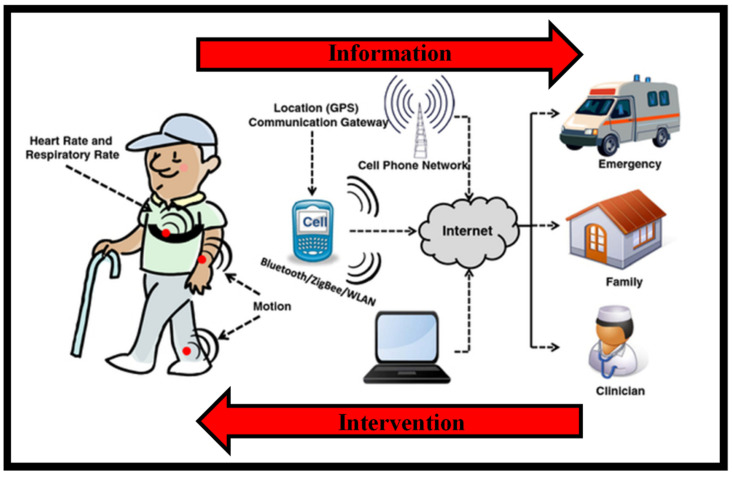
Design of a remote health observing arrangement established on wearables. Health-related data are collected via body-worn wireless devices and transferred to the caregiver via a data gateway, for instance, a mobile phone. Caregivers can utilize this data to execute interventions as required [19].

Figure 2 is a conceptual picture of a remote surveillance system [19]. Wearable sensors collect physiological and movement data, allowing patients’ status to be monitored. Sensors are employed in different ways depending on the therapeutic use of interest. When monitoring patients with congestive heart failure or COPD who are receiving clinical intervention, sensors for monitoring vital signs would be deployed. Sensors for recording movement data might be employed in applications monitoring the efficacy of home-based recovery programs in stroke survivors or in the usage of mobility assistance equipment among the elderly. Patients’ data are transmitted to a mobile phone or an access point through wireless transmission that is then relayed to a distant center over the internet. Data processing applied throughout the arrangement detects emergency circumstances, and an alert message is delivered to a trauma service center to give rapid aid to patients. Family members and caregivers are contacted in the event of an emergency, but they may also be informed in other instances, such as when the patient needs help taking his/her prescriptions. Clinical staff can remotely examine the patient’s condition and be notified if a medical decision must be taken.

Almost every analyte that a clinician could want to assess from a patient can now be measured using diagnostic instruments [23,24,25]. Unfortunately, such devices are not wearable, and blood draws and traditional bench-top analysis procedures are still required. Therefore, the key issue on many people’s minds is: how can wearable sensor automation begin to cross over into modalities that detect more precise physiological issues, such as: validating a child’s health while in the mother’s womb by tracking mechanical fetal motion; telling the difference between a deadly seizure and greater physical exertion; warning that an athlete or worker is becoming severely dehydrated; informing the health-conscious about how much overly refined white bread boosted their blood sugar levels; or mapping and controlling viral infection in a community before most of the population becomes symptomatic.

This review is devoted to recent advancements in wearable sensors established on optical phenomena. We tried our best to review two types of wearable sensors: (1) optical sensors powered by a battery; (2) battery-free skin-like sensors. The paper is structured in the following manner. In Section 2, the global market and demand for wearable optical sensors are discussed, which gives an insight into the importance of this topic. In Section 3, a brief description of the working mechanism of the unintrusive wearable optical sensors is presented. It has become quite simple to receive the body data by placing a compact sensing device on the body. In Section 4, the continuous battery-powered body surveillance devices are considered. This section has two sub-parts. In Section 4.1, recent developments and several types of commercially available pulse surveillance devices are reviewed. The place of wearing those devices on the human body plays a significant role in collecting precise data. In Section 4.2, recent advancements in continuous glucose surveillance devices are reviewed. Afterward, a new topic related to skin-like wearable sensors, which has emerged recently, is discussed in Section 5. These sensors are highly attractive, flexile, portable and do not require an integrated battery. The paper ends with final remarks in Section 6.

## 2. Demand for Wearable Optical Sensors

Even though both electronics and photonics are important in the prospect of wearables, this paper will concentrate on wearable optical devices. Optical sensors are expected to account for 13% of the wearable market by 2020, with optical and optoelectronic (OE) technologies also playing a role in other market segments, for instance chemical or elastic and pressure sensors. In recent years, several platforms have been used to produce optical sensors for refractive index sensing applications [26,27,28,29,30]. Optical sensors are distinct in that they are resistant to electromagnetic radiation, can probe nanoscale volumes, permit unintrusive examination of biological substance at comparatively deep penetration depths, and frequently use low-cost, water-, and corrosion-resistant sensing components. These resources have been used to detect and quantify ion, protein, and viral concentration, as well as pulse, blood pressure (BP), blood oxygenation, abdominal and thoracic respiration rate, targeted localized bending, and movement. Optical sensors, like all other sensing devices, must handle the magnified problems of proper signal-to-noise ratio (SNR), restricted dynamic range, signal specificity, and user variability in the setting of wearable devices. Furthermore, there is the issue of surrounding light interfering with signal readings, as well as poor light penetration into the skin and other bio-fluids, which is unique to optical sensing devices. New optical sensing elements and integration techniques, such as photonic textiles [31], innovative colorimetric [32] and fluorometric materials [33], and flexile photonics [34], are currently being researched to solve these difficulties.

**Figure 3:**
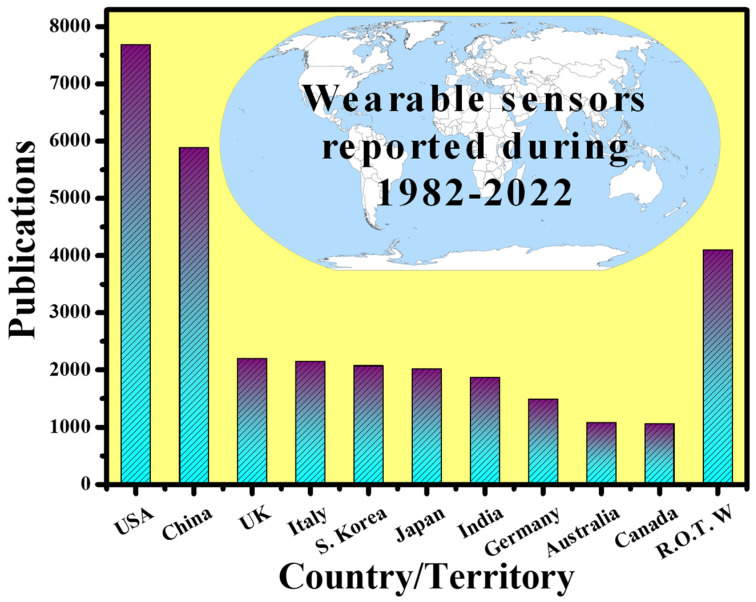
Scientific publications on “wearable sensors (electronics and photonics)” around the world. Results were collected on 19 November 2021.

By 2025, the worldwide wearable sensing industry is expected to be worth USD 5.5 billion, with currently developing technologies accounting for nearly a third of that total [35]. Over 1/10 Americans now possess a wearable sensing gadget, such as a specialized fitness surveillance device, a threefold increase from 2012 [36]. Fitness trackers and smartwatches can create personalized health profiles by gathering data on the pulse, blood oxygen level, movement, speed, step count, and even eating and sleeping patterns, using mobile phones and cloud connections [37]. Such gadgets are especially appealing for at-home health surveillance, particularly for the rising number of seniors who are living independently. Wearables can offer a reliable and thorough patient health record, minimize the resource load on hospitals, and expedite the reaction time in event of an emergency by empowering older users, their families, caregivers, and Medicare professionals, through remote health surveillance capabilities. Wearable technologies for elderly health surveillance are already making an effect, with total device shipments connected to wearable technologies for elderly health surveillance expected to achieve USD 44 million in 2019. Wearables are also becoming the latest means for medical practitioners and healthcare workers for hand-free, computationally assisted quick diagnoses and other health decision making, through ergonomic displays and voice control features. Wearables may also be employed to create augmented reality, for reasons such as better viewing of vital organs and tissue during surgery [38]. Wearable sensing device automation is even being employed for environmental surveillance. The capacity to simply monitor plant health, air quality, or toxins across a vast region via crowdsourcing is interesting, and certain new wearable devices make it even easier. Wearable sensing devices have been researched around the world. Figure 3 shows the number of publications indexed in the Scopus database published in the last four decades (1982–2022) concerning the countries. It can be seen that the USA is leading this research topic with the highest number of publications, which is two times higher than the rest of the world (R.O.T.W).

Recent advancements in manufacturing and packaging processes have supported the low-cost embedding of many OE devices and sensing devices on a chip. Furthermore, these chips are extensively employed in many kinds of physiological, biomechanical, and biological sensing, examining and gathering biomedical data remotely, thanks to a combination of wireless 3G and 4G technologies [39]. This has resulted in lower Medicare expenditure and enhanced continuous tracking of critical data, particularly for athletes, resulting in increased performance [2]. The development of 5G networks, paired with technologies such as the internet of things (IoT), machine learning, and artificial intelligence, will further revolutionize the future of remote biomedical sensing. Today, medical practitioners use portable diagnostic instruments, such as glucometers, which offer instantaneous data and are typically unintrusive or less intrusive [40].

However, automation for totally unintrusive blood sugar tracking is currently being developed. As a result, the main goal here is to build unintrusive wearable sensing devices for these sorts of diagnostics that may be employed in uninterrupted tracking procedures. Furthermore, the population is rapidly growing, and physicians are in limited supply all around the world. This has compelled the ordinary person to look for alternate choices that would help physicians use their time more efficiently. Individuals can use wearables to trace their important physiological data, with the option to visit a medical practitioner only when necessary [41]. The growing need for personal diagnosis and tracking is a robust sign of the benefits of wearables. Their application is not limited to tracking blood sugar levels; they have recently been anticipated as a substitute scheme for performing rapid HIV diagnoses [42,43], timely recognition of Alzheimer’s syndrome [44,45], and perspiration tracking via a wearable paper-based sweat sensing device [46], taking custom-made medicine to a new level [47]. Wearable interfaces, such as wearable electronics, electronic skin sensing devices, flexile displays, intelligent robotics, and implanted medical devices, have advanced rapidly in recent years [48]. Due to their unique structure, most of these devices are flexile, portable, adaptable, and easy to use, and they can even be directly bonded with human skin [37,49,50].

There are several non-implantable wearable sensors available for consumer health and medical research, and some devices are currently being used in regular clinical practice. Table 1 lists some of the most prevalent devices [51]. Mechanical, physiological, and biochemical sensors are the three basic types of sensors available. Sensors are available in a variety of grades, from consumer to clinical to research grade. The data from these sensors have been utilized for a variety of purposes, including tracking gait, diagnosing atrial fibrillation, and measuring blood glucose, to name a few.

**Table 1:** Examples of common consumer, clinical, and research-grade wearable sensors [51].

Manufacturer	Model	Market	Cost (USD)	Form Factor	Sensors	US FDA Status	Ref.
Abbott	Libre	Ambulatory diabetes monitoring	149.98 (cost for reader and 10-day sensor)	Semi-invasive	CGM	Approved	[52,53]
AliveCor	Kardia Band	Consumer	199	Wristband	ECG	Cleared	[54]
Apple	Watch Series 3	Consumer	329	Watch	Accel, ambient light sensor, BALT, Gyro, PPG HR, GPS	Pre-certified	[55]
Ava Science, Inc	Ava Wristband	Consumer	249	Wristband	Accel, EDA, PPG HR, Temperature sensors	Approved	[56]
Bloomlife	Smart Pregnancy tracker	Consumer (rental)	20/week	Abdominal patch	Accel, 3-channel AFE	-	[56]
Preventice	Bodyguardian Heart	Ambulatory cardiac monitoring	Ordered through physician, billed directly to insurance	Chest patch	Accel, EFG	Cleared	[57]
Oura	Oura ring	Consumer	299–999	Ring	Accel, Gyro, PPG HR, Skin temperature	-	[58]
Orpyx	Surro Gait Rx	Ambulatory gait monitoring	Ordered through physician	Watch, shoe insert, shoe pod	Pressure	-	[59]
Orpyx	Surro Sense Rx	Ambulatory gait monitoring	Ordered through physician	Watch, shoe insert, shoe pod	Pressure	Cleared	[60]
iRhythm	Ziopatch	Ambulatory cardiac monitoring	Ordered through physician, billed directly to insurance	Chest patch	ECG	Cleared	[61]
Medtronic	Enlite	Ambulatory diabetes monitoring	-	Semi-invasive	CGM	Approved	[62]

## 3. Working Mechanism of Unintrusive Optical Sensing Devices

Unintrusive biomedical measures are often conducted optically, with a light source of a certain wavelength (λ) being revealed to the area of the skin where the evaluation is desired [63]. The sensing device detects reflected and absorbed light, as well as refracted light, and then characterizes and quantifies the biological data (identical sensations as employed by spectrophotometer). When transmitting an optical signal through the skin, the λ is the most important component, since it controls how far the light can penetrate. Depending on the required penetration depth and substantial absorption peak for the relevant sensing application, the λ of these light sources can range from UV to deep IR. The detectors range from broadband photodiodes (PDs) to avalanche photodetectors and photomultiplier tubes. Several illustrations of related passive devices for light capture, λ selection, and light steering are integrated optics, diffraction gratings, narrowband optical filters, and bulk lenses.

**Figure 4:**
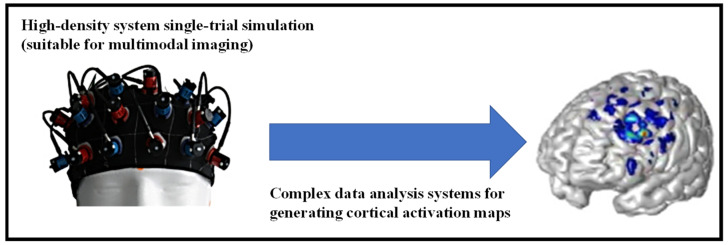
Multichannel fNIRS instrument.

The skin may also be employed as a window to see how the hidden organs are doing physiologically. The use of functional near-IR spectroscopy (fNIRS) to study oxygenation variations in the human brain is one such approach [64]. The current high-temporal-resolution multichannel systems simultaneously perform numerous measurements and display the findings in the form of a map or picture across a specified cortical area, employing three separate NIRS methods and complicated data analysis tools. The implementation of multichannel wearable/wireless devices that allow fNIRS measurements even during regular everyday activities represents the promise that exists for fNIRS more than for any other neuroimaging modality, as shown in Figure 4.

**Figure 5:**
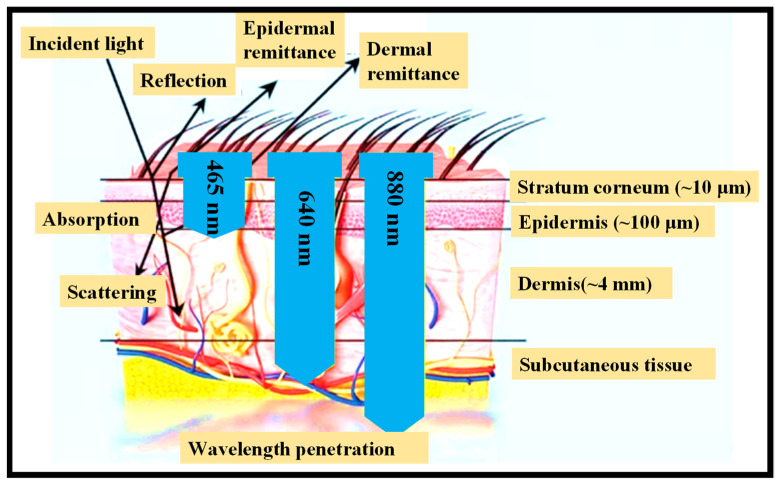
At different sites in the skin layers, incident light displays reflection, absorption, and scattering effects. In terms of λ, light penetrates into the skin [68].

Through its optical interfaces, the skin can sometimes provide a passive conduit for physiological data collecting from hidden vascular systems and organs. As a result, when constructing wearable optical sensing devices, it is critical to consider the skin’s optical characteristics [65]. The human skin’s absorption, transmission, and scattering may be studied by separating the skin into three layers with different optical characteristics:

(1) The stratum corneum, which is extremely keratinized owing to the presence of dead squamous cells.
(2) The hidden epidermis, which comprises skin pigmentation (mostly melanin) that absorbs shorter λ, such as UV, and visible (VIS) light to some extent [66].
(3) The dermis, which is extremely vascularized and can be described through VIS light and contains carotene, blood hemoglobin, and bilirubin [67]. Because of its thickness relative to the layers above, the dermis attenuates most of the VIS light, as seen in Figure 5.

Device geometries are determined by the application and evaluation sites on the skin. The light source is mounted opposite the detector in most hard-wired arrangements, as well as traditional wireless devices. This setup guarantees that the detected light interacts with the target tissue across a long optical channel length, resulting in high signal attenuation for pulsatile change extraction [69]. This geometry has the problem of being limited to relevant parts of the anatomy, such as the finger or ear lobe, and it does not provide a simple method for system downsizing [70]. Backscattered reflection approaches allow the light source and detector to be placed near one another in the same plane. As a result, evaluations may be taken via interfaces to practically any part of the body, with minimal downsizing and wireless operation.

Measurements in the reflectance mode, on the other hand, are prone to motion artefacts [71]. In this case, parasitic noise is created by tiny variations in the relative placement of the optical modules to the probing volume. In this context, digital and analog filtering techniques can be useful [72], and systematic compensatory methods that use accelerometers as motion sensors can offer considerable benefits, albeit at the cost of increased device complexity. As a result, traditional gear for PPG reflection mode measurements is often big and heavy, particularly when wireless operation and power supply are involved. There are additional difficulties in harmonizing the total power utilization and total size of the system with the measurement’s signal to noise ratio, where the device current for the light source and the distance between the source and the detector are essential characteristics [73].

## 4. Continuous Body Tracking Devices Powered by Battery

Optical sensing devices are now commercially available. The most popular optical sensing devices used in wearable electronics are for detecting blood oxygenation and tracking pulse [74]. The absorption and scattering characteristics of light concerning the location of the body describe each clinical or biological event when diagnosing it. The oximetry technique, which makes use of variations in the optical characteristics of hemoglobin in its deoxygenated and oxygenated state, is the most visible example [75]. The estimation of critical physiological characteristics, such as pulse inconstancy or variability using photoplethysmography (PPG) and oxygen saturation in arterial blood using pulse oximetry, is possible by utilizing analysis of the pulsatile component of the bloodstream [76,77].

Basic physiological signals include a lot of health-related data. Users are increasingly familiar with the concept of having every step, pulse, and breath recorded and analyzed to offer constant feedback on their health and everyday activities. As a result of this tendency, enterprises are under a lot of pressure to develop accurate, cost-effective, and durable continuous body tracking systems. Wearable optical sensing devices have thus far mostly been utilized for heart tracking [74,78]. PPG signals have been the focus of several wearable appliances. PPG signals are volumetric measures of an organ, most often subcutaneous blood vessels. The expansion and contraction of arterial volume owing to blood pumping can be observed by variations in optical absorption by lighting a perfuse region of the skin and tracking the reflected or transmitted signal. The pulse rate is consequently determined by the frequency of fluctuating optical absorption (i.e., the AC component of the PPG signal), and the amplitude of the AC component relates to BP in both the systolic and diastolic phases of the cardiac cycle. This method may also be used to test blood oxygenation by using two light λs with differing relative absorption by oxygen-loaded hemoglobin [79]. Revealing the mode of transmission when opposed to reflection mode, PPG sensing devices often generate a stronger signal with less motion artefact. However, transmission mode PPG sensing devices have a restricted signal site/location, since they must be in areas with high blood profusion, where optical signals have a possibility of travelling to the opposite side of the tissue and being recorded by the receiving PDs.

### 4.1. Heart Rate Monitors (HRMs)

PPG-based wearable optical heart rate monitors (HRMs) have grown extremely popular, with a slew of tech businesses and products being developed and marketed [80,81]. Sony, Microsoft, Apple, Motorola, FitBit, MioGlobal, and Masimo, among others, have all developed optical PPG sensing devices that may be worn on the wrist, around the chest, or even in-ear designs using headphone-based optical sensing devices that operate in reflection mode. Ring PPG sensing devices are also of interest because of their small size and potential to produce better signals via transmission-based measurements due to their position on the finger. Commercially accessible sensing devices now perform a wide range of optical and electrical tasks, including measuring PPGs for pulse surveillance, as well as blood oxygen levels, tracing the number of steps, breathing rate, temperature, and even estimating sleep quality [82]. Despite their economic success, there is much debate concerning the exact accuracy and dependability of these gadgets, particularly while performing high-intensity actions. There is no large-scale contributor, peer-reviewed studies that validate the correctness, motion artefact tolerance, and user-to-user variability of commercial wearable HRMs that we are aware of. The strength of the measured PPG signal is strongly location dependent, limiting the precision and repeatability of the wearable PPG device when consumers do not have the sensing devices securely or consistently attached, which is one of the causes for most of the disagreement [83]. Smith et al. proposed a checkboard-style architecture with alternating OLED and pin PD pixels to overcome this challenge [84]. The PPG signal would be recorded across the array of sensing devices upon startup, and the system would ultimately lock onto the sub-region within the checkboard with the sturdiest signal, reducing DC background noise by turning off OLED pixels not directly adjacent to the perfusion location liable for the robust signal [84].

Athletes are always looking for new technologies and cures to help them improve their health and performance [85]. Athletes are increasingly turning to wearable sensing devices to track their training and recuperation. Athletes’ internal and external workloads are presently monitored via wearable devices used by sports teams. The capacity to continuously monitor biomarkers from saliva or sweat in an unintrusive manner remains the next automation gap for sports medicine specialists, in order to personalize hydration and healing programs to the individual athlete [50]. Wearable sensing devices have evolved from a device to a system perspective during the last two decades, with the system combining the device with data. While earlier research focused on specific technical domains of the wearables field, such as sensing devices [86,87], materials [88,89], and soft interfaces [90], or on the production and application of such devices to focus on a particular medical condition, such as atrial fibrillation [91], cystic fibrosis [92], or diabetes [93,94], there is still an unmet medical requirement to evaluate, develop, and confirm this automation, particularly for sports medicine [95].

**Figure 6:**
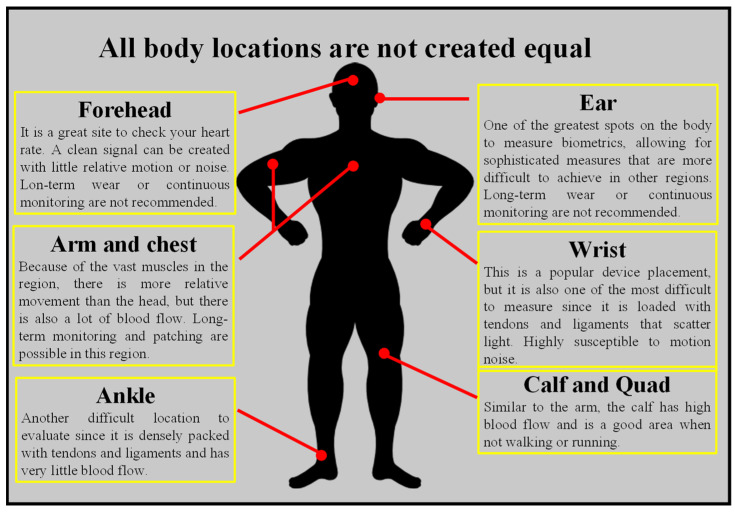
Different possible spots in the body to place HRM to obtain the best pulse.

Optical HRM functions by beaming the light into the body and detecting how light is scattered from blood flow. The technique works best in parts of the body where physiological factors that are not connected to blood flow restrict the quantity of light that is scattered or absorbed. When collecting data from parts of the body with a variety of tissues, such as bone, muscle, tendons, and so on, the accuracy may be reduced. Parts of the body undergo greater movement when the body is in motion, such as the wrists and ankles, which has a detrimental influence. There are different spots on the body where wearables can be worn to collect the best pulse, as shown in Figure 6.

**Figure 7:**
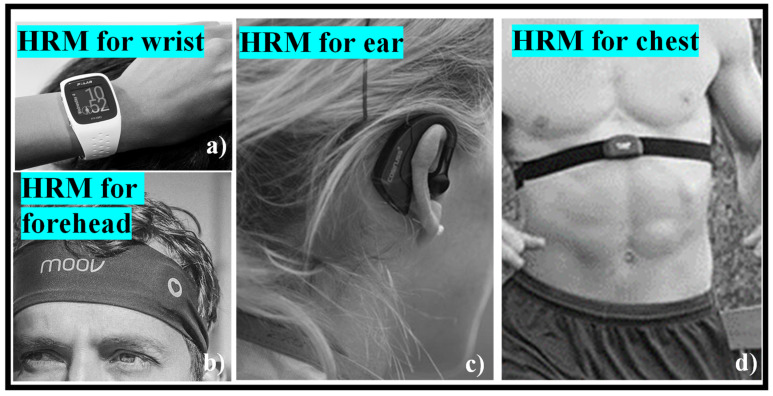
Commercially available HRM: (**a**) wrist [99], (**b**) forehead [100], (**c**) ear [101], (**d**) chest [99].

While power meters have established the gold standard for gauging effort on the bike, the best HRM may still be a useful addition to one’s toolkit. It can tell one how one’s body is performing and how it recovers from exertion on a climb or when that additional push is needed to keep a wheel in place. While the traditional chest band is still popular, optical HRMs have begun to gain traction, both as wrist-based sensing devices on the bottom of smartwatches and fitness trackers and as stand-alone devices. For a long time, HRMs necessitated the use of a chest band. Electrodes are put against the skin, and electrocardiography is utilized to record the electrical motion of the heart. HRM chest bands are remarkably accurate, regardless of price, with some devices, such as the Polar H10, claiming to track the pulse to a granular degree of precision. A chest band had a 99% correlation with an electrocardiograph, according to a research letter published in [96], while another study published in [97] revealed a Polar H7 to be 99.6% accurate when tested against an ECG. The pads require a little moisture to pick up the electrical pulses from one’s heart, so they may take a few minutes to generate an accurate reading after one starts sweating—alternatively, one can lick the electrodes before putting them on for quick reading, but we think that is a little strange. Many individuals have a love–hate relationship with chest-based HRMs, since the elastic band is unpleasant and can slip down during a ride. Most employ a detachable pod that holds the hardware to broadcast the ANT+ and Bluetooth signals, as well as a coin cell battery, accelerometers, gyroscopes, and sometimes a little memory to store running data and even entire activities. A few commercially available HRMs to be worn on different body parts are shown in Figure 7a–d.

Additionally, all-optical approaches for tracking the heart cycle in an MRI have been proposed. Rothmaier et al., for example, showed the application of photonic textiles for pulse oximetry measurements [98]. The researchers were capable of recovering a PPG in transmission mode when the finger was lit by a peripheral light source of λ = 690 nm and 830 nm by weaving polymer optical fibers (POFs) into the forefinger of a glove. The modified Lambert–Beer law was utilized to compute arterial oxygen saturation using this dual-λ light. By utilizing an amalgamation of coupling augmentation methods, for instance roughening the fiber surface, adding fiber back reflectors, and strategic fiber cuts in the direction of the incident light, it is possible to optimize the POF incorporation of diverse weaving and embroidered conformations to realize a nearly 100-time enhancement in coupling competences over the unaltered woven POF fibers. This unique technique is appealing, but it must still be enhanced in coupling effectiveness to obtain a high SNR in the PPG signal, as well as solve the same key issues related to motion artefacts that traditional PPG optical sensing devices encounter.

### 4.2. Continuous Glucose Monitoring (CGM)

Diabetes is a disorder that affects around 415 million people worldwide. The most prevalent kind of diabetes is type 1 diabetes, which occurs when the body attacks the cells in the pancreas, preventing it from producing insulin [102]. Type 2 diabetes, on the other hand, occurs when the body is incapable of generating enough insulin, or when the insulin that is produced is ineffective [103]. Although diabetics may live healthy, happy, and productive lives, maintaining blood sugar control is an important component of treating the disease. To do so, it is necessary to keep a close eye on the blood sugar levels. The advancements in medical automation have been astounding since the first blood sugar test strip was produced in 1965 [104]. CGM was established as a means of regulating blood sugar levels in 1999, and it was one of the most significant advancements in the history of blood sugar tracking. Since 1999, CGM has grown in sophistication, with new products always building on the development of previous ones. Flash continuous glucose monitoring (FCGM) is one of the most notable new kinds of CGM [105,106].

**Figure 8:**
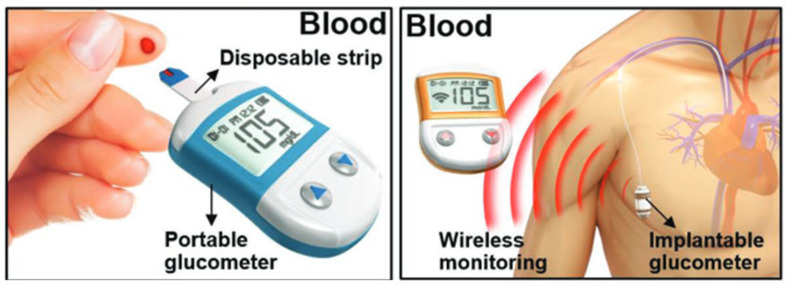
Invasive glucose monitoring systems [109].

Self-monitoring of blood sugar remains an extremely prevalent method of glucose monitoring (SMBG) [107]. This entails pricking the patient’s finger and drawing a little blood sample to determine the amount of blood sugar (Figure 8, left). Afterward, the patient is given insulin or glucose, as needed. This strategy, while simple, has certain disadvantages. Sufferers may find SMBG inconvenient, since testing might be uncomfortable. It is described as antisocial by many people, and it is influenced by daily living activities. This can be problematic, especially for younger individuals. Importantly, for this approach to be effective, regular testing is necessary, which reduces its attractiveness for consumers searching for discreet and simple ways to test their blood sugar levels [108]. An implantable glucose sensor is also used in clinics (Figure 8, right) [109]. The embedded sensor is more intrusive and sensitive to biofouling, but it provides CGM. Furthermore, because it detects glucose in the intravenous blood rather than the peripheral blood, an implanted sensor improves the accuracy and clinical usefulness of the data. Even though various blood-based monitoring methods have been devised, diabetic patients do not always follow the protocol due to the discomfort and difficulty connected with the intrusive detection procedure. In the case of type 1 diabetes, a considerable percentage of pediatric patients are particularly averse to the needle-pricking phase required for blood-based glucometers. It is critical that a painless and non-invasive approach for monitoring glucose levels be developed.

CGM, on the other hand, allows patients to monitor their blood sugar levels at all times using a wearable device that gathers data on the body’s blood sugar levels. CGM devices are available 24 h a day and incorporate alerts that sound when a patient’s blood sugar levels are abnormally high or low [21]. Finger pricking is no longer required or is much reduced. This is beneficial to the patient’s health and decreases the possibility of overestimation. Furthermore, CGM enables individuals to analyze data to spot trends and patterns, which may then be shared with their Medicare providers.

While research into novel technologies for miniaturizing electrochemical biosensors is still ongoing, other techniques established on optical sensing have become far more enticing recently [110]. Optical sensing technologies can circumvent many of the limitations associated with electrochemical sensing devices, such as sensing device sensitivity and stability being dependent on the enzyme utilized and interference with active substances (acetaminophen, ascorbate). Numerous optical recognition approaches have been proposed in the literature, including near-IR detection and Raman spectroscopy for unintrusive detection and fluorescence-based sensing devices for implanted systems [111]. Senseonics (Senseonics, Inc., Germantown, MD, USA) has recently successfully applied this last category of optical sensing technologies to develop the Eversense sensor, a fully implanted CGM system [112] that provides real-time blood sugar measurements through an external coupled transmitter for an expected lifetime of 6 months [113]. With a lifespan of 90 days and an accuracy of 11.4% MARD, the Eversense CGM is currently only certified for usage in European nations (CE mark acquired in 2016) [114]. While biocompatibility and patient acceptance of the new completely embedded automation remain major concerns, the approach’s primary virtues are its longevity and ease of use.

The sensing devices (which sit in the skin), the transmitter (which sits above the skin), and the receiver (either a smartphone or a handheld device that receives blood sugar data) are the three main components of most CGM sensing devices. The majority of CGM sensing devices (very thin wire-like components) are implanted under the skin (body fat) [115]. Typically, the sensing device is implanted with a tiny needle, using a simple at-home application device. The needle retracts almost immediately, leaving the sensing device in situ. The transmitter, which rests on top of the skin and is held in place by an adhesive, is the most visible part of a CGM. CGM sensing devices can be put in a variety of locations on the body, depending on the user’s preference: back of the arm, side/front torso, lower back, buttocks area, outer thigh, and so on. Areas where the body bends a lot naturally or where clothes can irritate or be a nuisance should be avoided. Inserting the CGM sensing device is a simple and almost painless procedure.

A blood sugar sensing device has been designed that is both cost effective and extremely wearable, with a short data-gathering time frame that allows for an unintrusive, long-term CGM system [116]. During the variation of blood sugar concentration at the wrist tissue, the innovative biosensing device exploits unique data of the pulsatile to continuous components of the arterial blood volume pulsation. The combined VIS–NIR spectroscopy was used to measure the reflected optical signal.

**Figure 9:**
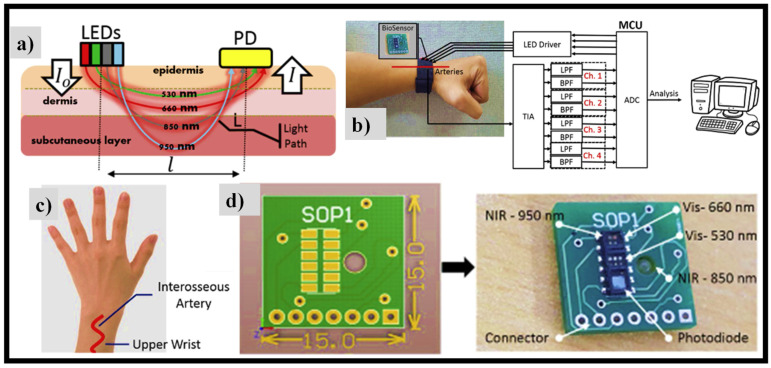
(**a**) Diffused reflectance PPG signal in multiple λ measurements [116]. Prototype system: (**b**) block diagram of analog signal processing [116], (**c**) sensing device wear location on the wrist [116], (**d**) the overall sensing device design on PCB [116]. Reproduced with permission from [116]. Elsevier B.V., 2019.

NIR light may penetrate multiple layers of the skin and reach arteries in the subcutaneous tissue, as illustrated in Figure 9a, but VIS light, such as green and red light, can only make it to the capillaries and arterioles in the dermis tissue. Consequently, by collecting reflected light in different optical λs at varying levels of blood sugar concentration, several spectra data may be acquired that can be used for multivariate analysis. As shown in Figure 9a, NIR light can penetrate multiple layers of skin to reach the arteries in the subcutaneous tissue, but VIS light, such as green and red lights, can only reach the capillaries and arterioles in the dermis tissue [116]. As a result, by collecting reflected light in various optical λs at various levels of blood sugar concentration, we will obtain a variety of spectral data that can be used in multivariate analysis. As illustrated in Figure 9b, the suggested prototype acquires data via analog signal filtering.

The sensing device system is affixed to the wristband’s rear side and is in direct touch with the subject’s skin tissue. It is situated between the interosseous arteries on the subject’s outer wrist, with an overall sensing device size of 15 × 15 mm^2^, as shown in Figure 9c,d, respectively. An SFH7060 (OSRAM semiconductor Inc., Germany) multichip sensing device package with dimensions of 7.2 × 2.5 × 0.9 mm is used to transmit IR LED light of 950 nm, red LED light of 660 nm, and green LED light of 530 nm, and to identify the reflected light utilizing an integrated PD with a spectral range of 400 nm to 1100 nm. Furthermore, for 850 nm, a VSMY2853 GIR LED (Vishay Intertechnology Inc., USA) with a 100 mA forward current is employed. As a result, two VIS LEDs and two NIR LEDs are employed in total.

The median association coefficient between the approximate and reference blood sugar concentration was 0.86, with a standard prediction error of 6.16 mg/dL, in an in vivo experiment including 12 participants in a 2 h controlled carbohydrate-rich diet. Furthermore, the suggested sensing device’s dependability was tested for a whole day. The results revealed that the model constructed the day before could accurately predict a full-day blood sugar concentration that was measured the next day.

## 5. Skin-like Wearable Sensing Devices

In the biomedical investigation, clinical treatment, and remote diagnostics of health state, accurate tracking of temperature, BP, oxygenation, blood flow, and electrophysiology is critical [117,118]. Most commercially available wearable gadgets are mechanically hard components attached to bands and worn on the wrist, with form factors eventually constrained by the size and weight of the batteries required for the power supply. Wearable gadgets with “skin-like” qualities are a new kind of automation that is only starting to make its way out of research labs and into pre-commercial prototypes [119,120]. The use of near-field communication (NFC) automation for multicolor light emission and recognition in a way that permits accurate evaluation of the optical features of the skin—to detect peripheral vascular disease (PVD) and assess coloration—and/or of color-responsive materials for environmental discovery, is demonstrated in several battery-free, wireless OE devices.

### 5.1. Skin–Electronics Interface

Wearable sensor integration must consider not only flexible and biocompatible materials but also intimate contact forms on the human epidermis. Here, three types of skin interfaces are examined: tattoo-like, medical bands, and textiles.

(i). Tattoo

Tattoo-like wearable sensors represent an emerging class of ideal device shapes with their conformal and practically invisible look. Many critical signals, such as the electrocardiogram (ECG), electromyogram (EMG), and electroencephalogram (EEG), can be accurately monitored using tattoo-like sensors, since intimate physical contact may be obtained [121]. In addition, chemical sensing is available in this format. Tattoo-based non-invasive lactate monitoring was proven in a typical application by analyzing sweat during cycling activity with an enzymatic biosensor. A lightweight and breathable kind of on-skin electronics that bonded tattoo-like conductive nanomesh directly onto human skin was presented to improve gas permeability and wearing comfort [122]. Tactile sensing was studied for a long time without generating inflammation.

(ii). Band

Alternative technologies for on-skin applications are required for sophisticated sensor systems that include stiff components, such as integrated circuit chips. Medical bands are frequently utilized to make direct touch with the skin without causing undue pain. To continually monitor the pulse wave of the radial artery, a polymer transistor-based pulse sensor may be readily fixed on skin using a standard bandage [123]. A completely integrated wristband with a chemical sensor array and FPCB was recently created for in situ detection of sweat metabolites and electrolytes [124]. Similarly, a graphene-based band platform applied to the forearm was used to monitor glucose levels in the interstitial fluid in real time [125].

(iii). Textile

Wearable textile sensors can be sewn onto clothes or knitted into them for long-term use. Conductive textiles may be used to make functional electrical components, including antennas, resonators, and pressure sensors [126]. Sensor textiles for skin interfaces can be developed as clothing or gloves. Textile wearable devices are commonly used as an appropriate platform for energy-harvesting applications owing to frictions with the human body. Clothing with a triboelectric energy generator (TENG) and supercapacitors have recently been developed [127]. Energy collecting and storage were shown using a natural arm-swinging action while walking. With skin interfaces, the main goal is to create close skin contact and capture high-quality sensory data. Rigid electronics create gaps between the device and the skin, resulting in artefacts and noise while moving. Meanwhile, many tattoo-based sensors still require additional power and analysis devices, and more research combining low power consumption and totally flexible skin electronics is underway [128].

Smart textiles are becoming a popular technique for wearable sensor systems because they provide greater transparency between the sensor and the user, i.e., the sensor system is light weight, small, and does not obstruct the user’s movement. The use of compact and integrated sensors in smart textiles, as well as their benefits of easy installation and removal, improves the system’s use. Considering these benefits, advances in flexible electronics have paved the way for the creation of flexible wearable sensors. Smart textile technologies continue to point to even greater downsizing, low energy consumption, and wireless connectivity, all of which are in line with IoT device needs. Resistive sensors embedded in fabric patches and twin core microfibers for capacitive measurements, as well as other embedment techniques and electronics, are examples of such advancements.

One of the rising trends of our time is the transformation of textiles from things that shield humans from temperature, rain, and other elements into useful fabrics with extra capabilities. These so-called smart textiles frequently include electronics that are integrated to varying degrees to create unique designs, connect jackets to smartphones, track firefighters, make automatic emergency calls by avalanche victims, or detect biosignals that are particularly important for athletes, the elderly, and ill people who need to be monitored for longer periods of time. Aside from the sensors, connectivity, a power supply, and a data processor are also required, with varying degrees of integration. While full integration of sensors and extra electronics into textile materials is not always possible currently, the benefits of this method are obvious. Electrodes that come into direct contact with skin can be made from more skin-friendly materials and in more pleasant designs than, for instance, conventional ECG electrodes or the somewhat inflexible chest straps used in sports pulse measurements. A long-term ECG based on textile electrodes and textile-integrated electronics is significantly more comfortable than the still prevalent variant with rigid equipment, many cables, and adhesive electrodes due to the additional capability of embedding all essential cables into the textile.

### 5.2. Materials and Structural Designs

The mechanical qualities of wearable sensing devices must be soft, flexile, and compliant with curved skin for them to be integrated with the human body. Silicon is the most common semiconductor material, although its modulus is 1 × 10^5^ times greater than that of human skin. Given the significant mechanical mismatch, the most logical solution is to produce naturally soft and conductive materials to provide mechanical compatibility and increased wearing comfort. Soft electronic materials for skin applications have a lot of potential because of recent advances in material science. However, there are still issues with these newly produced materials. While liquid conductors [129], such as gallium metal alloys, are low in toxicity and activity and can give unlimited stretchability, most liquid conductors require microfluidic channels to deliver elastomer for skin sensing devices [130]. Although hydrogels [131,132] and polymers [133] are very biocompatible, their effectiveness is restricted by their poor conductivity, and many hydrogels dry up and harden with time. To improve performance and stability, current biocompatible materials are mainly made up of composite materials that include hydrogels, polymers, and nanoparticles [134]. To avoid any health risks, caution should be exercised when employing nanomaterials, such as carbon nanotubes encapsulated inside elastomers [135,136]. Biocompatible materials need to be developed further for improved stability and breathability in practical applications [137].

Polydimethylsiloxane (PDMS), polyethylene terephthalate (PET), polyurethane (PU), polyimide (PI), polyethylene naphthalate (PEN), polyethylene oxide (PEO), polystyrene sulfonate (PEDOT: PSS), Ecoflex, and other materials are commonly used as flexile metal foils or non-metallic substrates in flexile skin-like sensing devices [138]. Various polymers were found to be appropriate for use as flexile substrates in the creation of flexile devices.

**Figure 10:**
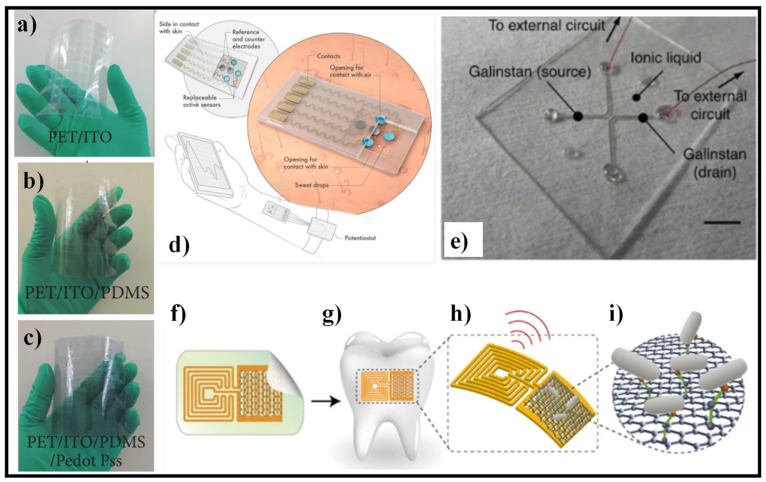
Flexible substrates for biosensors, (**a**–**c**) PDMS- and PEDOT:PSS-based pressure sensor [139], (**d**) wearable sweat sensor based on MXene [143], Reproduced with permission from [143]. John Wiley & Sons, Inc., 2019, (**e**) fully fabricated liquid heterojunction device, consisting of an ionic liquid active channel and GaInSn source/drain electrodes [144], (**f**) graphene-based wireless nanosensor [145], (**g**) biotransfer of the nanosensing architecture onto the surface of a tooth [145], (**h**) magnified schematic of the sensing element, illustrating wireless readout [145], (**i**) binding of pathogenic bacteria by peptides self-assembled on the graphene nanotransducer [145].

The use of PDMS and transparent electrodes is proposed for a flexible pressure sensor, as shown in Figure 10a–c. The whole device has an 82% transmittance and a minimum bending radius of 18 mm. Apart from that, the impact of annealing temperature on the mechanical characteristics of PDMS is discussed in [139]. The results demonstrate that the PDMS film has acceptable compression properties but poor dynamic responsiveness at a lower annealing temperature of 80 °C. The compression property of PDMS films is dramatically diminished at higher temperatures. The annealing temperature of around 110 °C is determined to be the optimal compromise between compression property and dynamic responsiveness for PDMS film. The pressure sensor has an excellent sensitivity of 0.025 kPa^−1^ and a strong response property when cured at 110 °C. The invention paves the way for intelligent transparent sensing applications in the future.

Furthermore, MXene-based hybrid materials with superior characteristics have a lot of potential in next-generation pressure sensing devices for a wide range of applications [140]. The fast growth of graphene and other 2D material syntheses, as well as new manufacturing methods for functional devices, have alleviated some of the problems associated with traditional materials, allowing for the application of emerging wearable flexile electronics and complex functional products [141]. The chemical structures and characteristics of illustrative biopolymers, which outlined the design and production strategies for biocompatible conductors established on these biopolymers, are demonstrated, and highlighted production methods for several biocompatible conductors for flexile bioelectronics [142].

Lei et al. created a wearable biosensor based on MXene for sweat analysis, as shown in Figure 10d [143]. The device was created by combining 2D MXene (Ti_3_C_2_T_x_) nanosheets with a Ti_3_C_2_T_x_/PB composite and enzyme to create an oxygen-rich enzyme biosensor for H_2_O_2_ detection. Ti_3_C_2_T_x_/PB composites demonstrated higher electrochemical performance toward H_2_O_2_ detection than carbon nanotubes/PB and graphene/PB composites due to the high conductivity and strong electrochemical activity of exfoliated MXene. The sensing device has a changeable sensor component that may be inserted and replaced with customized sensors to track various analytes, such as glucose, lactate, or pH value. The device’s substrate is made of superhydrophobic carbon fiber, which was utilized to establish a tri-phase contact and protect the connection against sweat corrosion. Artificial perspiration was used to test the as-prepared device’s sensing capabilities. In addition, the sensor was put to a test on human participants and was utilized to analyze perspiration. The results revealed great sensitivity and reproducibility in simultaneous glucose and lactate readings.

Electronic systems may be utilized in a conformal manner on curved and soft surfaces thanks to mechanically deformable devices and sensors. Because liquids are intrinsically more flexible than solids, sensors that use liquids enclosed in soft templates as the sensing component provide an appropriate platform for such applications. However, because of the difficulties in fabricating liquid-based junctions due to intermixing, liquid-based devices have been confined to metal lines based on a single liquid component. A stable foundation for producing liquid–liquid heterojunction devices paves the way for liquid-state electrical systems to become a reality. The device design and manufacturing strategy are general for various detecting liquids, allowing for the demonstration of sensors that respond to various stimuli. Temperature, humidity, and oxygen sensors have been developed as proof of the concept, employing various ionic liquids, displaying great sensitivity and outstanding mechanical deformability due to the intrinsic characteristic of the liquid phase, as shown in Figure 10e [144].

Researchers created a biotransferrable graphene wireless nanosensor for bacteria detection in saliva [145]. The sensor’s design is shown in Figure 10f. A graphene-based sensing element with a wireless readout coil was first created on silk fibroin. Second, the graphene-based transducer was biotransferred onto a tooth surface before the supporting silk film was dissolved, as depicted in Figure 10g. After that, self-assembling antimicrobial peptides were applied to the graphene monolayer to increase detection specificity. When the target bacterium is recognized and bound, the graphene film’s electroconductivity is modified and wirelessly monitored using an inductively connected radio frequency reader device (Figure 10h,i). The created hybrid nanosensor was shown to have a low detection limit (100 CFU/mL), operating without batteries, and being capable of distant wireless sensing.

### 5.3. Communication and Data Analysis

Near-field communication (NFC) is a new form of automation that permits two electronic devices to communicate by merely placing them close together [146]. For chloride detection, for instance, NFC-supported thermography was paired with colorimetric microfluidic sensing devices [147]. An NFC chip was coupled with an onboard temperature sensing device to provide real-time sweat loss and electrolyte concentration diagnostics. NFC transmission, for example, permits not only data but also power transfer. A hybrid skin-interfaced device with electrochemical, colorimetric, and volumetric sweat analysis capabilities was recently introduced [148]. A tiny NFC electronic module and a microfluidic system make up the battery-free arrangement. The system is started by bringing an NFC-supported portable device close enough to capture the wireless, real-time data, as well as digital photos for colorimetric analysis [148]. Despite its light weight and low power utilization, NFC automation has an extreme delivery distance of about 4 inches and requires the antenna to be kept in a specific orientation, as well as a proximal power source placed close to the subject, limiting the subject’s range of activity and making it unsuitable for long-term use.

Sweat can be used to provide unintrusive, in situ biochemical tracking of physiological state, which might lead to new kinds of Medicare diagnostics and individualized hydration methods. Recent developments in sweat collecting and sensing automation are attractive; however, they are ineffective in extreme circumstances, such as aquatic or arid environments. Eliminating interference/contamination from surrounding water, maintaining robust adhesion during viscous drag forces and/or vigorous motion, and limiting the evaporation of collected perspiration all pose distinct problems. The materials and design of waterproof, epidermal, microfluidic, and electrical devices that stick to the skin, store, and analyze sweat, even while fully submerged, are discussed [147].

**Figure 11:**
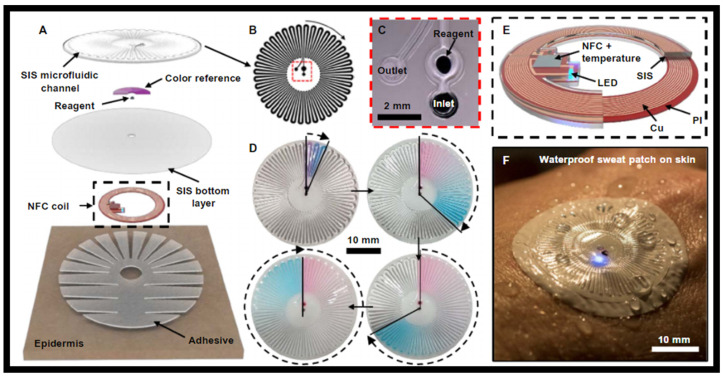
Skin-like microfluidic/electronic device that is waterproof, (**A**) schematic depiction of the major layers of a typical device [147], (**B**) geometry of microfluidic channels [147], (**C**) optical micrograph of the colorimetric reagent and microfluidic intake and outflow ports [147], (**D**) a flow-driven color shift is produced by a dye made up of blue and red water-soluble particles that dissolve at different speeds. The total volume of collected perspiration is calculated by counting the number of turns of filled tubes [147], (**E**) NFC coil for wireless evaluations of skin temperature [147], (**F**) sweat gathering in aquatic environments without contamination is supported by small outlet geometries and constituent polymer materials that are hydrophobic and largely impermeable to water and water vapor. Underwater functioning of the electronics, containing the NFC coil, integrated circuit chip, and indicator LED, is supported by dip-coating an encapsulation of the same material [147].

The device platform is made up of a waterproof combination of skin-like, or epidermal, microfluidic, and electronic arrangements that are laminated onto the skin for sweat collection, storage, and chemical analysis, as well as digital skin temperature evaluation in both aquatic and dryland environments, including the transition between the two. Micro-channels, a chamber containing a colorimetric chemical reagent, electronics for wireless communication and precise temperature tracking, a set of reference color markers, and a skin-safe glue are all included in the structures, as shown in Figure 11A [147]. The molded layer of poly (styreneisoprene-styrene) (SIS) attaches to a thin, flat sheet of SIS to define the sealed microfluidic arrangement and enclose the color markers, as shown in Figure 11B [147]. The colorimetric reagents are either a food dye for visual assessment of the degree of sweat filling of the μ-channel or a silver chloranilate solution that interacts with sweat to give a colorimetric response proportional to the chloride concentration. This reagent is kept in a compartment next to the intake, where it reacts when perspiration enters the channel, as shown in Figure 11C [147]. As the device fills with perspiration, the food dye comprises red and blue water-soluble particles with differing dissolving rates, resulting in a volume-dependent color gradient, as shown in Figure 11D [147]. A wireless interface to NFC-supported devices for transmitting digital identification codes and reading skin temperature is formed by a flexile magnetic loop antenna, a set of NFC components, and a LED as a mode for user notification. The NFC chips are encased in SIS, which permits a long-term operation even when the gadget is immersed in water, as shown in Figure 11E [147]. The LED emits light through the microfluidic layers during wireless operation in a wet environment, as shown in Figure 11F [147].

For wearable Medicare, Bluetooth low energy (BLE) provides an alternate communication platform [149]. In comparison to NFC, BLE offers a quicker communication rate and a reading distance of over 30 feet, making it ideal for multipurpose sensing and long-term use. Integrated devices comprising of a rechargeable battery, a microcontroller, signal-processing circuits, and a Bluetooth transceiver were constructed on a flexile printed circuit board (FPCB) and coupled to a flexile electrochemical sensor in a typical configuration of an electrochemical sensor arrangement [124]. Bluetooth captures data in real time, sends them to the user’s phone, and then uploads them to cloud servers. The mechanical rigidity of a BLE module, on the other hand, is undesirable for skin interfaces, and most commercially available BLE modules consume milliwatts of power, implying high power needs.

The frequency of RF transmission is set at 13.56 MHz, and it achieves coupling between an initiator and a passive target [150]. A chip-free and battery-free on-skin sensor network that uses RF identification automation to communicate wirelessly with flexile readout circuits mounted to textiles is reported in [151]. To establish a conformal skin contact, the elastic sensors were made of inherently soft materials without any hard silicon circuits or batteries, whereas the initiator circuit incorporated a Bluetooth transceiver for data transfer to a smartphone. The device was shown to continually track pulse, respiration, and body movement. Wireless communication and data analysis advancements have permitted increased shrinking and incorporation of wearable sensor arrangements; however, significant restrictions remain. NFC and RF communication can be made completely flexile and conformal to the skin, but they have a small range of operation and need antenna-readout electronics to be positioned near the sensors. BLE permits a quicker and more efficient data transfer, but it requires specific integrated circuit chips that cannot be produced flexibly at this time. Because wireless communication modules consume a significant amount of power, they must be paired with power management to provide a fully integrated sensor platform.

### 5.4. Applications of Skin-like Sensors

Several battery-free, wireless OE devices are demonstrated that utilize near-field communication (NFC) automation [152] for multicolor light emission and recognition in a way that permits specific evaluation of the optical attributes of the skin—to detect PVD and estimate coloration—and/or of color-responsive materials for environmental recognition [153]. Explicit cases comprise devices that can monitor pulse, tissue oxygenation, pressure pulse dynamics, UV exposure, and skin color through an integrated collection of time-multiplexed miniaturized LEDs and photodetectors whose signals are amplified and digitized before wireless broadcast [154,155]. Form factors proficient in soft and conformal lamination onto the skin, as well as the capacity to operate effectively under substantial strain (up to 30% uniaxial) deformation, are the result of carefully optimized materials and mechanical designs. These findings point to a basis for automation that may be used in both Medicare and non-Medicare applications. These systems are significant advancements over devices that rely on wired data broadcast and traditional power sources to evaluate arterial blood oxygenation using commercial or organic LEDs and photodetectors mounted on flexile rather than elastic supports [98,156].

Flexible skin-like sensing devices have accomplished numerous functionalities previously inaccessible for standard sensing devices due to their deformability, lightness, portability, and flexibility. Physical, chemical, physiological, and multifunctional flexile skin-like sensing devices are the four primary kinds of flexile skin-like sensing devices, depending on the quantities they detect. Physical sensing devices are created by utilizing the physical characteristics of the substances that are susceptible to the measured quantities [157]. Chemical sensing devices are made up of responsive components that transform chemical quantities into electrical quantities, such as the composition and concentration of chemical compounds. Physiological sensing devices are sensing devices that detect and recognize biological and physiological quantities in living organisms by using different biological and physiological features or characteristics of biological substances. Pressure, strain, and temperature are the three most common physical variables that may be sensed by flexile skin-like physical sensing devices, which can transform external physical stimulus signals, such as pressure, strain, and temperature into electrical impulses, completing the skin’s sensing function [158]. Multifunctional sensing devices are made up of several distinct kinds of sensing devices combined into a single unit that can identify and test multiple variables instantaneously [159]. It has been stated, for example, that the multifunctional electronic skin can monitor pressure, tension, and temperature all at the same time [160]. Flexile sensing devices have a wide range of categories and uses, thus worldwide research on them has accelerated in recent years [158].

**Figure 12:**
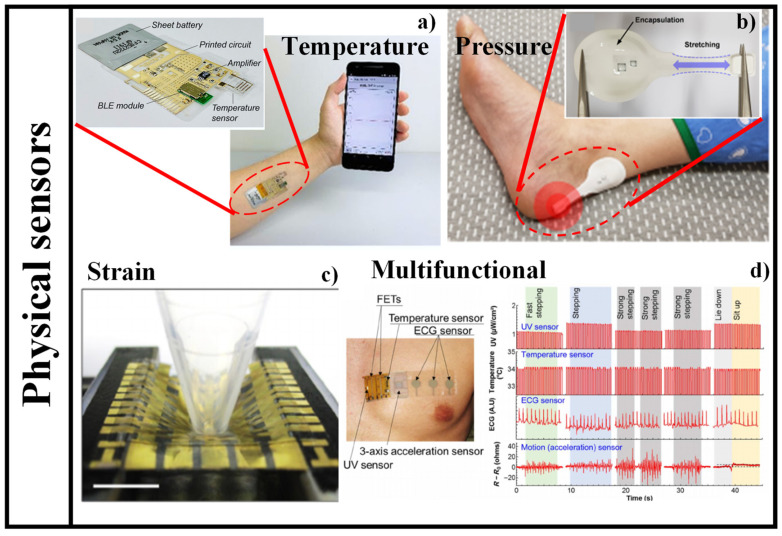
Different applications of flexile physical sensing devices, (**a**) graphical illustration of wireless sensing platform affixed to an arm for real-time body temperature tracking. Inset shows the optical image of the wireless temperature sensing platform with printed temperature sensing device [161], (**b**) photograph of a device affixed to body heel that is susceptible to pressure. Inset shows the photograph of a battery-free, wireless pressure sensing device [165], (**c**) photograph of strain-sensitive elastic active-matrix transistor array as skin-like elastic strain sensing device [166], (**d**) image of the multifunctional device attached directly onto the skin (**left**) real-time acceleration (motion), ECG, skin temperature, and UV tracking results [167].

A novel design of a temperature sensing device is proposed, which is made of cross-linked poly (3,4-ethylenedioxythiophene): poly (styrenesulfonate) (PEDOT: PSS) [161]. Significant improvements in humidity stability and temperature sensitivity of PEDOT: PSS-based film were achieved by combining the crosslinker (3-glycidloxypropyl) trimethoxysilane (GOPS) with the fluorinated polymer passivation (CYTOP). The fabricated sensor device showed outstanding stability in a range of ambient humidity from 30% to 80% relative humidity, as well as high sensitivity of −0.77%/°C for temperature detection between 25 °C and 50 °C. The schematic diagram of a wireless sensing platform mounted on an arm for real-time body temperature tracking is shown in Figure 12a [161].

The most often utilized sensing devices for body detection are strain and pressure sensing devices. With countless papers, the discipline is fast evolving and prospering [162,163,164]. The mechanism, structural compositions, performance quantities, and applications of pressure and strain sensing devices vary widely. A new kind of sensing device system is presented that is soft and may be worn on the skin [165]. A pressure-responsive element established on membrane deflection is included in the design, as well as a battery-free, wireless mode of operation that allows for multisite evaluations around the body. From a pair of main antennas positioned beneath the mattress and coupled to a wireless reader and a multiplexer located at the bedside, such devices provide continuous, simultaneous pressure and temperature evaluations in a sequential readout scheme. Bench-top evaluations and numerical simulations of essential aspects are part of the detection device’s and system’s experimental assessment. Two hemiplegic patients and a tetraplegic patient participated in clinical studies to establish the automation’s practicality, usefulness, and long-term stability in operational hospital settings. Figure 12b shows the photograph of a device mounted on a body heel that is susceptible to pressure [165].

To address the expectations for future electronic skin application, skin-like sensing devices should be elastic and self-healing. Despite recent significant breakthroughs in skin-inspired electronic materials, imparting these required capabilities to an active semiconductor remains difficult. The combination of a polymer semiconductor and a self-healing elastomer, both of which are vigorously cross-linked by metal coordination, results in a strain-sensitive, elastic, and autonomously self-healing semiconducting film [166]. The blend film became strain sensitive when the percolation threshold of the polymer semiconductor was controlled, with a gauge factor of 5.75 × 10^5^ at 100% strain in an elastic transistor. The composite film is also highly elastic and independently self-healable at room temperature. The ability to detect strain distribution through surface deformation was proven using a fully integrated 5 × 5 elastic active-matrix transistor sensing device array. The photograph of strain-sensitive elastic active-matrix transistor array as a skin-like elastic strain sensing device is shown in Figure 12c [166].

Real-time Medicare tracking may help forecast and prevent illnesses, as well as enhance treatment by detecting diseases early on. To permit continuous tracking of a person’s health, wearable, comfortable sensing devices are necessary; additional significant aspects of this automation are device elasticity, affordable components, management, and multifunctionality. To meet these requirements, a flexile, multipurpose printed Medicare sensor, with a three-axis acceleration sensing device to detect body movement and motion, is exhibited [167]. The device features a modular design with two detachable modules, one of which is non-disposable and the other of which is disposable and designed to be worn in contact with the skin. This disposable sensing sheet’s design considers hygiene concerns, as well as low-cost materials and assembly procedures. It also includes integrated, printed sensing devices to monitor temperature, acceleration, and electrocardiograms, as well as a kirigami structure that allows for skin stretching. The device’s reusable component incorporates more costly components, as well as a UV light sensing device controlled by carbon nanotube thin-film transistors and a physically flexile and secure liquid metal contact for linking to the disposable sensing sheet. After determining the electrical characteristics of the transistors and flexile sensing devices, a pre-commercial device capable of Medicare tracking, as well as physical activity detection, is proposed, demonstrating that this device is an outstanding podium for the expansion of commercially feasible, wearable Medicare monitors. Figure 12d (left) shows the image of the multifunctional device attached directly to the skin. Moreover, the real-time acceleration (motion), ECG, skin temperature, and UV tracking outcomes are also shown in Figure 12d (right) [167].

## 6. Final Remarks

Life expectancy has risen steadily in most countries over the last several decades due to considerable advances in medicine, public health, and individual and environmental cleanliness. Nevertheless, rising life expectancy combined with dropping birth rates is likely to result in a huge elderly demographic shortly, putting a major strain on these countries’ socio-economic structures. As a result, developing cost-effective, easy-to-use solutions for seniors’ Medicare and well-being is critical. Remote health tracking, established on unintrusive and wearable sensors, actuators, and current communication and data technologies, is a cost-efficient option that permits the elderly to remain in their homes rather than expensive Medicare institutions. These devices will also permit Medicare workers to track key physiological signals of their patients in real time, analyze health problems, and offer feedback remotely. Owing to unintrusive sampling and high precision, wearable sensors may analyze the abnormal conditions of the physical or chemical components of the body in real time, exposing the body state in real time. Most commercially available wearable devices are mechanically hard modules linked to bands and worn on the wrist, with form factors ultimately limited by the size and weight of the power source batteries. Wearable devices with skin-like characteristics are a relatively new form of automation that is only now making its way out of research laboratories and into pre-commercial prototypes. We presented recent advances in battery-powered wearable sensors established on optical phenomena, market demand, working mechanism, and several commercially available wearable optical sensors. Moreover, skin-like battery-free sensors were also presented in this study, which represents a significant advancement in wearable sensing automation. Several battery-free, wireless OE devices demonstrate the use of near-field communication automation for multicolor light emission and recognition in a way that permits accurate evaluations of the optical characteristics of the skin—to detect PVD and evaluate coloration—and/or of color-responsive materials for environmental detection. Due to their deformability, lightness, mobility, and flexibility, skin-like sensors have achieved several functions previously unavailable to traditional sensors. Flexile skin-like sensors may be classified into four types based on the quantities they detect: physical, chemical, physiological, and multifunctional.
